# Driving CARs with alternative navigation tools – the potential of engineered binding scaffolds

**DOI:** 10.1111/febs.15523

**Published:** 2020-08-31

**Authors:** Charlotte U. Zajc, Benjamin Salzer, Joseph M. Taft, Sai T. Reddy, Manfred Lehner, Michael W. Traxlmayr

**Affiliations:** ^1^ Christian Doppler Laboratory for Next Generation CAR T Cells Vienna Austria; ^2^ Department of Chemistry Institute of Biochemistry BOKU‐University of Natural Resources and Life Sciences Vienna Austria; ^3^ St. Anna Children’s Cancer Research Institute Vienna Austria; ^4^ Department of Biosystems Science and Engineering ETH Zurich Basel Switzerland; ^5^ Department of Pediatrics St. Anna Kinderspital Medical University of Vienna Austria

**Keywords:** CAR T cells, DARPin, diabody formation, immunogenicity, monobody, nanobody, protein engineering, rcSso7d, scFv clustering, tonic signaling

## Abstract

T cells that are genetically engineered to express chimeric antigen receptors (CAR T cells) have shown impressive clinical efficacy against B‐cell malignancies. In contrast to these highly potent CD19‐targeting CAR T cells, many of those directed against other tumor entities and antigens currently suffer from several limitations. For example, it has been demonstrated that many scFvs used as antigen‐binding domains in CARs show some degree of oligomerization, which leads to tonic signaling, T cell exhaustion, and poor performance *in vivo*. Therefore, in many cases alternatives to scFvs would be beneficial. Fortunately, due to the development of powerful protein engineering technologies, also non‐immunoglobulin‐based scaffolds can be engineered to specifically recognize antigens, thus eliminating the historical dependence on antibody‐based binding domains. Here, we discuss the advantages and disadvantages of such engineered binding scaffolds, in particular with respect to their application in CARs. We review recent studies, collectively showing that there is no functional or biochemical aspect that necessitates the use of scFvs in CARs. Instead, antigen recognition can also be mediated efficiently by engineered binding scaffolds, as well as natural ligands or receptors fused to the CAR backbone. Finally, we critically discuss the risk of immunogenicity and show that the extent of nonhuman amino acid stretches in engineered scaffolds—even in those based on nonhuman proteins—is more similar to humanized scFvs than might be anticipated. Together, we expect that engineered binding scaffolds and natural ligands and receptors will be increasingly used for the design of CAR T cells.

## Graphical abstract

A large body of evidence has accumulated, demonstrating that there is no biochemical or functional requirement for the use of scFvs in CARs. Instead, highly potent CARs have been constructed based on several engineered non‐immunoglobulin‐based binding scaffolds. This review also includes a critical discussion on the risk of immunogenicity associated with different types of engineered binding domains and humanized or human scFvs.

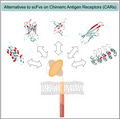

**Abbreviations**
CAR: chimeric antigen receptor
scFv: single‐chain variable fragment
DARPin: designed ankyrin repeat protein
FN3: 10th type III domain of human fibronectin
TanCAR: tandem CAR
EGFR: epidermal growth factor receptor
HER2: human epidermal growth factor receptor 2
hRBP4: human retinol‐binding protein 4

## Introduction

**Fig. 1:**
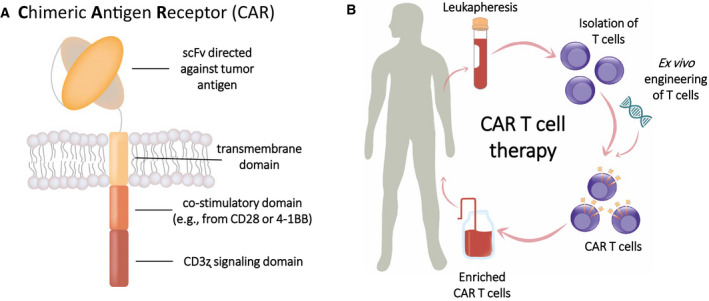
(A) Structure of a standard second‐generation CAR, consisting of an scFv targeting a tumor‐associated antigen, a transmembrane domain, and intracellular signaling domains derived from a costimulatory receptor and CD3ζ. (B) Basic scheme of CAR T cell therapy. Leukocytes are harvested by leukapheresis from the patient, followed by isolation of T cells and genetic engineering to induce CAR expression. After expansion, enriched CAR T cells are administered to the patient.

Immunotherapy for the treatment of cancer has experienced a breakthrough in the last decade. One of the most promising approaches in this field is CAR T cell therapy, that is, the adoptive transfer of T cells genetically engineered to express chimeric antigen receptors (CARs) [1, 2]. The standard CAR molecule combines an extracellular antigen‐binding domain with intracellular signaling domains, which activate the T cell in response to antigen recognition. The most commonly used CARs consist of an extracellular single‐chain variable fragment (scFv) that is directed against a tumor‐associated antigen and usually derived from murine, humanized or human antibody sequences, followed by a hinge or spacer region, a transmembrane domain and intracellular signaling domains derived from a costimulatory receptor (usually CD28 or 4‐1BB) and from CD3ζ (Fig. 1A). Very briefly, the manufacturing of CAR T cells involves isolation of T cells from the patients' blood, transduction with a lentivirus or retrovirus carrying the CAR gene, *ex vivo* expansion, and infusion of the final CAR T cell product into the patient (Fig. 1B). The remarkable clinical responses of patients treated with CAR T cells targeting the antigen CD19 expressed on B cell acute lymphoblastic leukemia (B‐ALL) and B cell lymphoma led to FDA approval of two CD19‐targeting CAR T cell products in 2017: Kymriah (tisagenlecleucel) and Yescarta (axicabtagene ciloleucel) [3]. Despite the highly promising clinical outcomes achieved by these products, CAR T cells frequently cause adverse events such as neurotoxicity and the release of large amounts of cytokines, resulting in a condition termed cytokine release syndrome [4, 5]. Furthermore, in contrast to the treatment of B cell malignancies, such high potency of CAR T cells has rarely been observed with solid tumors. Thus, there is still considerable room for improvement of this relatively young therapeutic approach. Since the CAR molecule is assembled from several domains, each of these components may be independently optimized to yield CAR T cells with improved therapeutic properties [6]. Moreover, the right components need to be matched to yield an efficient CAR molecule. For example, depending on the targeted epitope (proximal or distal to the membrane), different spacer lengths between the antigen‐targeting domain and the transmembrane domain may be required to yield an optimal distance between the T cell and the target cell membranes [7, 8, 9, 10].

In this review, we focus on the antigen‐binding domain of CAR molecules. We discuss potential limitations of scFvs and advantages of engineered binding scaffolds as new options for CARs. We give an overview of the most commonly used binding scaffolds based on either nonhuman or human proteins and highlight those which have already been used within a CAR. Finally, we critically discuss the potential immunogenicity of engineered binding scaffolds in comparison with that of humanized and 'fully human' antibody fragments.

## Limitations of scFvs as antigen recognition domains on CARs

**Fig. 2:**
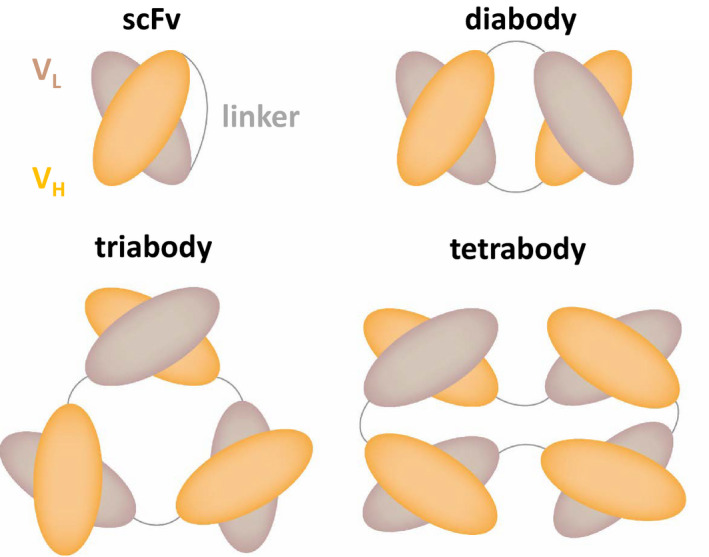
Schematic representation of a conventional scFv and its oligomeric states, which are formed by all scFvs to various extents, depending on linker length and the specific scFv variant, among other factors. These oligomerization effects may occur both in a soluble format and when scFvs are integrated into CARs.

In the vast majority of CARs currently under preclinical and clinical investigation, scFvs are used as the antigen‐targeting domain. Indeed, scFvs can be very attractive because of their small size and the possibility to engineer them to bind virtually any target molecule accessible on the cell surface. In addition, antibodies are already available for most antigens, facilitating the rapid and inexpensive generation of scFvs [11]. However, the major disadvantage of scFvs is their dependency on correct pairing between the linked V_H_ and V_L_ domains. This domain architecture entails the risk of uncontrolled heterodimerization between two scFv molecules. That is, instead of forming a correct V_H_‐V_L_ pair within an scFv molecule, a V_H_ can also pair with the V_L_ of a neighboring molecule and vice versa, thereby forming so‐called diabodies [12] (Fig. 2). Depending on the length of the peptide linker, even complexes of three or four cross‐paired scFvs can be formed [13] (Fig. 2). This tendency of scFvs to oligomerize is well established in the protein engineering field and has been demonstrated not only for isolated soluble scFvs [12, 13, 14], but also for scFvs fused to a crystallizable fragment (Fc) [15] and, importantly, for scFvs integrated into CARs ([16] and [17]). This phenomenon can be explained by the lack of the natural structural support from the C_H_1‐C_L_ interface that is normally part of a full‐length antibody and/or by partial unfolding at the V_H_‐V_L_ interface, ultimately resulting in domain swapping [18]. Alternatively, mispairing may simply be a result of a stochastic process during protein folding, where a given V_H_ may randomly pair either with its intramolecular V_L_ partner or with a neighboring V_L_ from another molecule. Apart from mispairing, scFvs have extended hydrophobic patches that are normally not solvent‐exposed in a full‐length antibody, potentially causing aggregation in the absence of domain swapping [18, 19].

When incorporated into CARs, oligomerization of scFvs and subsequent CAR clustering can be detrimental since it can lead to antigen‐independent constitutive signaling of the CARs, which is referred to as tonic signaling [16, 18, 20]. Long and colleagues observed such tonic signaling in CAR T cells harboring an scFv against the disialoganglioside GD2, which subsequently led to T cell exhaustion and thereby poor *in vivo* performance of the GD2‐specific CAR [16]. This effect was further accompanied by lower CAR T cell expansion *in vitro* and increased T cell apoptosis. Importantly, the authors additionally investigated CARs containing four other scFvs, of which three induced tonic CAR signaling and T cell exhaustion at varying degrees: two different CARs recognizing CD22 (scFvs HA22 and m971) and one CAR directed against HER2 (scFv 4D5). Only the clinically used and highly potent CD19‐directed CAR (scFv FMC63) did not show constitutive activation and T cell exhaustion, which may at least partially explain the success of CAR T cells based on this scFv in the clinics. Taken together, these studies highlight that tonic signaling can be observed at varying degrees with many commonly used scFvs and can impact the performance of CAR T cell therapy.

In line with the functional data discussed above, recent experiments in our laboratories provided direct biochemical evidence that scFvs indeed induce CAR clustering on the T cell surface [17]. Briefly, we showed that CARs based on strictly monomeric backbones were dimerized/oligomerized by a low‐affinity version of the HER2‐directed scFv 4D5, resulting in multivalent antigen recognition and thereby avidity‐based activation of this low‐affinity CAR. Of note, co‐expression of two separate membrane‐anchored constructs comprising the V_H_ and V_L_ of 4D5, respectively, resulted in a functional CAR, but eliminated the clustering‐induced avidity effects. Thus, these data strongly suggest that the removal of the linker between the V_H_ and V_L_ prevented diabody formation, CAR clustering, and avidity‐based activation of the otherwise monomeric low‐affinity CARs.

**Fig. 3:**
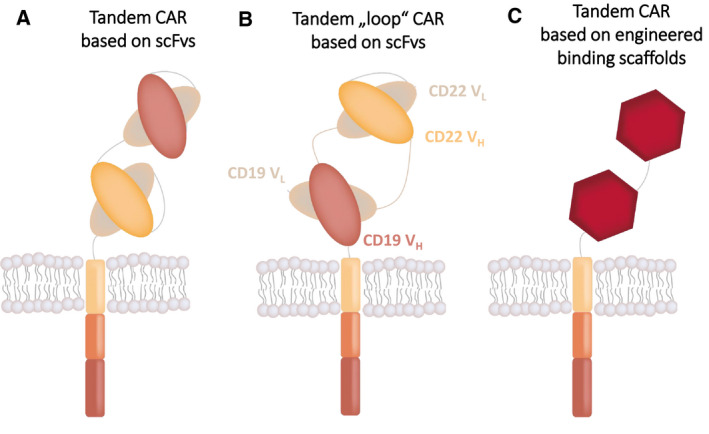
Schemes of (A) a tandem CAR based on two scFvs, (B) a tandem 'loop' CAR having a special configuration of the CD19 and CD22 scFv (CD19 V_L_‐CD22 V_H_‐CD22 V_L_‐CD19 V_H_), and (C) a tandem CAR based on two engineered binding scaffolds.

Potential mispairing problems are expected to be even more pronounced in so‐called tandem CARs (TanCARs), in which two scFvs are expressed in tandem (i.e., fused to each other) to yield a bispecific CAR (Fig. 3A) [21, 22, 23]. Such TanCARs have mostly been constructed with the goal to design CARs with OR gate function. That is, those TanCARs recognize target cells expressing antigen A, or antigen B or both, thereby strongly reducing the risk of resistance due to antigen loss. While the construction of TanCARs containing two scFvs is definitely feasible, extensive optimization may be required to avoid mispairing between different scFvs (either those within a TanCAR or between neighboring TanCAR molecules). For example, the generation of a CD19‐CD22 bispecific CAR required a loop configuration, in which the CD22 scFv is expressed in between the V_H_ and V_L_ of the CD19 scFv (CD19V_L_‐CD22V_H_‐CD22V_L_‐CD19V_H;_ Fig. 3B) [24, 25, 26]. Although this creative design was highly successful, resulting in a potent CAR that is currently being evaluated in clinical trials, it nevertheless demonstrates that the construction of TanCARs based on two scFvs is a particularly challenging task. In contrast, the construction of a TanCAR based on two engineered single‐domain binding scaffolds is more straightforward, since this strategy precludes undesirable domain swapping and oligomerization (Fig. 3C).

## The potential of engineered binding scaffolds as alternatives to scFvs in CARs

Specific interaction of the CAR molecule with the target antigen and subsequent induction of T cell effector functions such as cytokine secretion and cytotoxicity is an essential prerequisite for effective CAR T cell therapy. An ideal antigen‐binding domain of a CAR molecule should meet the following requirements: (i) It should recognize its target molecule with high affinity and specificity, (ii) it should be stable and well expressed by primary human T cells, (iii) it should not induce CAR clustering and tonic signaling, and (iv) it should be as close as possible to human germline‐encoded proteins to minimize the risk of immunogenicity (immunogenicity will be extensively discussed below). While scFvs typically fulfill the first two requirements, many scFvs suffer from their tendency to induce tonic CAR signaling [16], as discussed above.

**Fig. 4:**
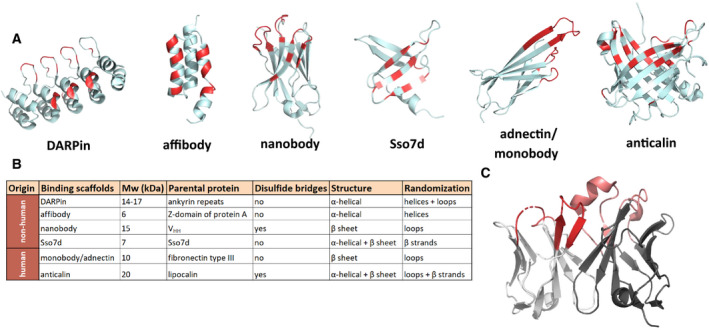
(A) Structures of the most commonly used engineered binding scaffolds derived from the Protein Data Bank (PDB) (DARPin: 4JB8 [107], affibody: 2KZI [108], nanobody: 5F1K [109], Sso7d: 1SSO [110], monobody/adnectin 3QWQ [111], and anticalin: 1L6M [112]). Randomized positions within the respective scaffolds are highlighted in red. (B) Overview of the key features of depicted binding scaffolds. (C) Complementarity‐determining regions (CDRs) in an scFv. The V_L_ domain is colored in light gray, the V_H_ domain in dark gray, and the respective CDRs are colored in dark and light red for the V_L_ and V_H_ domains, respectively. The depicted scFv is directed against IL‐15 (PDB: 2XQB). All graphics were generated using pymol.

Of note, the development of various display technologies, such as phage display [27, 28], yeast display [29, 30, 31], and ribosome display [32, 33], allows the generation of artificial binding sites also in non‐immunoglobulin‐based protein scaffolds. In other words, the emergence of these protein engineering technologies eliminated the historical dependency on antibody‐derived constructs for antigen recognition. This resulted in the generation of many different types of engineered binding scaffolds, which can be considered as non‐antibody‐based alternatives to scFvs. Engineered binding scaffolds are typically developed by randomly mutating a certain surface area on a stable protein domain, followed by the selection of suitable binders from the resulting library by using one of the display methods mentioned above. Numerous different engineered binding scaffolds have been described in the literature (reviewed in Ref. [34, 35]). They are derived from various organisms (human, bacteria, archaea, plants, or even artificially designed proteins), cover a broad variety in their architecture (i.e., α‐helical or β‐sheet proteins, or combinations thereof), vary in their sizes, and comprise completely different structural elements on which the engineered binding surface is located (e.g., flexible loop regions, or rigid structures based on α‐helices or β‐sheets) (Fig. 4A,B). In some scaffolds, the randomized positions in the loop regions resemble the complementarity‐determining regions (CDRs) within antibodies (Fig. 4C) with an overall similar global fold, as exemplarily illustrated for monobodies (Fig. 4A). Notably, alternative binding scaffolds harbor several advantages: (i) They are typically highly stable and well‐expressed; (ii) many scaffolds do not contain any disulfide bonds (in contrast to scFvs), which makes them applicable in the reducing environment present in the cytoplasm [36]; and (iii) they are usually composed of only one protein domain. The latter is the most important advantage of engineered binding scaffolds with respect to their application in CARs, since the single‐domain architecture precludes any mispairing issues as is observed with scFvs. As mentioned above, numerous engineered binding scaffolds have been described in the last two decades. Since the focus of this review is directed toward the application of those engineered proteins in CAR T cells, only a representative selection of binding scaffolds (particularly those that have been used for the generation of CARs) will be discussed below and presented in Fig. 4. For a more comprehensive overview of engineered binding scaffolds, we refer the reader to other reviews [37, 38, 39].

### Examples for binding scaffolds based on nonhuman proteins

#### Designed ankyrin repeat proteins

Among the best‐known alternative scaffolds are designed ankyrin repeat proteins (DARPins). DARPins comprise several structurally similar repeats that are based on a consensus sequence derived from several natural ankyrin repeat proteins [40, 41]. Depending on the number of repeats, DARPins are approximately 14–17 kDa in size. The engineered binding surface is composed of both surface residues on α‐helices and loop residues (Fig. 4A). DARPins have been selected for binding to a wide range of targets and can exhibit high affinities in the picomolar range while at the same time providing the advantage of high thermostability [42, 43, 44]. Further, these compactly folded scaffolds can even be fused to each other without impairing folding, stability, or specificity. As an example, this allowed the generation of bivalent DARPin fusions targeting two different subdomains on HER2, thereby leading to enhanced receptor inactivation [45]. Another multispecific DARPin targeting VEGF and hepatocyte growth factor (HGF), while also binding to human serum albumin (HSA) for increased half‐life [46], is currently tested in combination with a tyrosine kinase inhibitor in a clinical trial for patients with non‐small cell lung cancer (NCT03418532).

#### Affibodies

Affibodies are extremely small (approx. 6–7 kDa) protein scaffolds derived from the Z‐domain of *Staphylococcus aureus* protein A (Fig. 4A). In contrast to antibodies, whose binding surface is based on loop regions, the engineered binding site on affibodies is located on rigid α‐helices [34, 38, 47]. Affibodies have been selected against various targets and HER2‐ and IL‐17A‐specific affibodies are currently in clinical testing (NCT03655353 and NCT03591887, respectively) [34, 39, 48, 49].

#### Sso7d and Sac7d

Sso7d is another minimalist binding scaffold with a size of only 7 kDa (Fig. 4A). This DNA‐binding protein derived from the hyperthermophilic archaeon *Sulfolobus solfataricus* is free of cysteines and extremely thermostable (*T*_m_ of 99°C [50]). In addition to Sso7d, its close homolog Sac7d (derived from *Sulfolobus acidocaldarius*) has also been used for the selection of highly stable recognition domains [51]. Sso7d and Sac7d have been engineered to bind to completely different types of target molecules, including a small organic molecule (fluorescein), a peptide fragment (derived from β‐catenin) [52], and various proteins such as human Fc [53], among many others.

Recently, Traxlmayr et al. generated libraries based on a charge‐reduced version of Sso7d (reduced charge Sso7d, rcSso7d) to diminish unspecific interactions with mammalian cell surfaces resulting from excess positive charges on this DNA‐binding protein [50]. The resulting rcSso7d libraries were selected for binding to various antigens, such as human epidermal growth factor receptor (EGFR) [50] and for specific recognition of an oncogenic, mutated variant of human K‐Ras [36]. Recently, rcSso7d was also engineered to recognize human retinol‐binding protein 4 (hRBP4) in a small molecule‐dependent manner [54]. This ultimately yielded a molecular switch that enabled the functional control of CAR T cells by administration of an orally available small molecule, as will be further discussed below.

#### Nanobodies

Camelids are among the very few animals which express antibodies composed of only a heavy chain. As a consequence, the binding sites in those camelid antibodies only comprise a single domain, that is, the variable domain of the heavy chain (V_HH_). Separate expression of these V_HH_ domains yields single‐domain antibody fragments (~15 kDa) that are commonly referred to as nanobodies (Fig. 4A) [55]. Because the light chain is missing in nanobodies, they do not require additional folding steps as is required for scFvs, where the V_H_ and V_L_ need to assemble. Thus, while nanobodies are antibody‐derived, they possess the critical advantage of a single‐domain architecture, similar to most non‐antibody‐derived scaffold proteins.

Another benefit of nanobodies is their ability to reach some epitopes which are not accessible to conventional antibodies [56]. A good example for such a binding mode is nanobodies selected against the glycoprotein 140 of human immunodeficiency virus (HIV), which have been shown to neutralize the majority of known HIV strains [57]. The authors speculated that the longer CDR3 loop can bind to a neutralizing epitope, which normally is inaccessible to conventional antibody formats. Currently, several bispecific nanobodies are investigated in clinical trials (NCT03384745, NCT03468426, and NCT03972150). In early 2019, the first nanobody termed caplacizumab was approved by the FDA. Caplacizumab is a bivalent agent comprising two identical humanized nanobodies targeting von Willebrand factor, a key protein for blood coagulation. This protein is not only the first nanobody to be ever approved, but it is also the first therapy for patients with acquired thrombotic thrombocytopenic purpura, which is a rare disease characterized by excessive blood clotting [58].

### Examples for binding scaffolds based on human proteins

#### Monobodies/Adnectins

Monobodies—also termed adnectins—are small (10 kDa) and cysteine‐free scaffolds based on the 10th type III domain of human fibronectin (FN3) [59, 60]. They possess a β‐sandwich structure and an engineered binding surface that is typically based on three solvent‐exposed loops, resembling the CDR loops of antibodies (Fig. 4A) [59, 60, 61]. In an alternative library design, the binding site has been engineered on the 'side' of the FN3 domain, that is, including a β‐sheet and two‐loop regions (termed 'side‐and‐loop library'), thereby forming a concave binding surface [61].

Due to the human origin and high stability of the FN3 domain (*T*_m_ of 86°C [62]), monobodies have evolved as some of the most commonly used non‐antibody‐based scaffolds. Among the many monobodies generated so far, CT‐322 is among the most advanced products. It targets human VEGFR‐2, is attached to polyethylene glycol (PEG) to increase its half‐life, and was already tested in a phase II study for patients with recurrent glioblastoma which resulted in a partial response in at least one patient [63]. Alternatively, due to the lack of disulfide bonds (as is the case for most other binding scaffolds), monobodies have also been used for blocking various intracellular molecules and signaling pathways [64, 65].

#### Anticalins

Anticalins are engineered scaffolds based on the lipocalin family, of which 15 members have been described in humans [66]. Those proteins are approximately 20 kDa in size and have a characteristic eight‐stranded β‐barrel fold, which binds to small hydrophobic ligands such as fatty acids and vitamins [67, 68]. The four structurally flexible loops at the entrance of the binding pocket can be engineered to yield antigen‐specific anticalins (Fig. 4A) [69]. This has been used to generate anticalins directed against many different antigens for a broad spectrum of applications, involving therapeutic approaches and *in vivo* imaging as well as diagnostics [70]. Importantly, anticalins targeting VEGF‐A, PCSK9, hepcidin, IL‐4‐Rα, and a bispecific product targeting HER2 and the costimulatory receptor 4‐1BB have entered the clinical development for the treatment of a diverse set of diseases [71].

### Examples for engineered binding scaffolds used as antigen recognition domains in CARs

Despite the availability of a range of different binding scaffolds, the vast majority of CARs has been constructed based on scFvs. Nevertheless, over the last years several studies have accumulated, in which alternative binding scaffolds were successfully used as antigen recognition domains in CARs.

One example is a CAR in which a HER2‐specific DARPin was integrated into a CAR backbone of the second‐generation comprising intracellular signaling domains derived from CD28 and CD3ζ. This DARPinCAR could be expressed in T cells at levels comparable to that of a CAR based on a HER2‐specific scFv and mediated lysis of various HER2‐expressing human and murine tumor cell lines [72].

In another study, two HER2‐targeting DARPins were integrated into CARs comprising signaling domains derived from CD28, 4‐1BB, and CD3ζ [73]. In *in vitro* assays, the resulting DARPinCARs showed slightly higher capacities to induce T cell effector functions than an scFv‐based control CAR. Surprisingly, when tested in a mouse model, only one out of two DARPinCARs and the scFv‐CAR showed efficient tumor clearance. Interestingly, both the functional DARPinCAR and the scFv‐CAR target the same membrane distal epitope on HER2 while the other DARPin was shown to bind to the membrane‐proximal domain IV of HER2, suggesting that the location of the epitope plays an important role independent of the used binding domain [73].

De Munter et al. designed a bispecific CAR targeting both HER2 and CD20 by linking two llama‐derived nanobodies. Both bispecific and monospecific nanobody‐based CARs (termed nanoCARs) were expressed at levels comparable to that of a monospecific scFv‐based CAR. In addition, target cells engineered to express either HER2 or CD20, as well as those expressing both antigens, were efficiently lysed by the respective nanoCAR T cells [74]. This study is a good example, where the use of single‐domain binding scaffolds avoided the problem of mispairing—even though two antigen‐binding modules were expressed in series in one CAR molecule.

Xie et al. [75] generated nanobody‐based CAR T cells to target the tumor microenvironment. They selected nanobodies specific for either programmed death‐ligand 1 (PD‐L1) or the fibronectin splice variant EIIIB, an immune checkpoint and a marker of tumor extracellular matrix, respectively. Both the PD‐L1‐ and the EIIIB‐specific nanoCAR T cells efficiently delayed tumor growth in a B16 melanoma model.

Apart from DARPins and nanobodies, also monobodies have been applied for the design of CAR molecules. Monobodies, which had previously been engineered to bind to EGFR with low nanomolar affinities [76], were incorporated into CARs harboring two costimulatory domains. In general, the expression levels of monobody‐based CARs in primary T cells were comparable to that of an scFv‐based control CAR [77]. The EGFR‐specific monobody showed slightly lower affinity than the scFv derived from the EGFR‐specific clinical antibody cetuximab. The authors argued that this difference in affinity could be used for better discrimination of EGFR‐high‐ vs. EGFR‐low‐expressing cells, since it is known that high‐affinity CAR T cells recognize also healthy tissue expressing low antigen levels. Indeed, target cells with low levels of EGFR were still recognized by the scFv‐based CAR T cells while being spared by the monobody‐CAR T cells [77]. In contrast, target cells expressing high levels of EGFR were efficiently lysed by both types of CARs.

In a recent study, we engineered two completely different binding scaffolds (rcSso7d and monobodies) to recognize hRBP4 only when loaded with an orally available small molecule drug called A1120. That is, selected binders bound to the drug‐loaded conformation of hRBP4 with high affinity while exhibiting approximately 500‐fold lower affinity to hRBP4 in the absence of this small molecule [54]. This yielded a molecular ON switch, in which a protein–protein interaction can be turned on with an orally available small molecule drug. By incorporating one of these engineered molecular switches into a CAR, the assembly of a functional CAR molecule could be regulated by administration of the drug A1120. This ultimately enabled the functional control of primary human CAR T cells *in vitro*, as demonstrated by regulated cytokine secretion and cytotoxic activity.

Finally, in another study from our group rcSso7d‐based binders against EGFR and affibody‐based binders against HER2 were used to analyze the relationship between CAR dimerization, affinity of the antigen‐binding domain, and CAR T cell activation. This analysis was only enabled by the availability of those monomeric single‐domain binding scaffolds. In contrast, when we used an scFv directed against HER2, it was not possible to investigate the effect mediated by CAR dimerization, since dimerization/oligomerization was already induced by the scFv in an uncontrolled manner, as discussed above [17].

Taken together, these studies underline the versatility of engineered binding scaffolds for the design of CARs. Thus, there is no functional requirement that necessitates the use of scFvs as antigen‐binding domains in CARs. Instead, the suitability of an antigen recognition domain will be dictated by its recognized epitope, its affinity and specificity, its tendency to dimerize/oligomerize, and its expression rate when fused to a CAR backbone. In some cases, the optimal choice will be an scFv, whereas in other instances an engineered binding scaffold might be more suitable.

## Potential immunogenicity

Apart from the biochemical and functional properties of engineered binding scaffolds, their potential immunogenicity is another important factor that needs to be considered. Especially for binding scaffolds derived from nonhuman proteins, the risk of being recognized by the patient's immune system is a potential drawback. Indeed, it was already observed in early clinical studies that the administration of CAR T cells can induce both a humoral and cellular immune response, which leads to blockade and limited persistence of the CAR T cells [78]. Interestingly, patients, who relapsed with antigen‐positive tumor cells after treatment with anti‐CD19 CAR T cells based on a murine scFv, could be successfully treated with CAR T cells based on either a humanized anti‐CD19 scFv [79] or a 'fully human' anti‐CD22 scFv. This observation suggests that the rejection of the CAR T cells based on the murine scFv could be overcome by using scFvs that are closer to human germline sequences. Therefore, the CAR T cell field is moving toward the use of humanized or 'fully human' scFvs [25, 79, 80].

However, it is important to note that every engineered binding domain—even those derived from human sequences—is not 'fully human'. That is, in order to generate an antigen‐binding site, a number of surface positions need to be mutated. Even our immune system modifies the sequences of the variable domains of antibodies through junctional diversity [81] and somatic hypermutation [82] in order to achieve the high affinities that are typical for antibodies. Thus, apart from assembling the antibody genes from germline‐encoded V, D, and J segments, B cells undergo molecular processes to introduce additional diversity, thereby deviating from the germline sequence.

The risk of T cell‐mediated immunogenicity of a given polypeptide sequence depends on many factors, including protein/peptide processing efficiency, altered processing by the immune proteasome, recognition by a T cell receptor (TCR), and—most importantly—whether the resulting peptide is efficiently displayed on any of the major histocompatibility complex (MHC) alleles expressed in a given patient (or a patient population) [83, 84]. Several assays have been developed to predict—or at least estimate—the risk of immunogenicity, including *in silico* tools, *in vitro* models with peripheral blood mononuclear cells, and *in vivo* models using transgenic mice or nonhuman primates [84]. In case a certain sequence stretch in a protein is predicted to be potentially immunogenic, it is possible to reduce the immunogenicity of this epitope by inserting mutations. For example, the number of predicted T cell epitopes in green fluorescent protein (GFP) and *Pseudomonas* exotoxin A has been successfully reduced without compromising protein function [85, 86]. However, while those prediction models may help to reduce the risk of immunogenicity of lead candidates, a definitive answer on the immunogenicity of an engineered therapeutic protein will only be obtained upon testing in a reasonably sized patient population (covering a representative set of MHC molecules) [84].

As already mentioned, in addition to T cell‐mediated immune responses, B cell‐mediated immunity may also play a role. For example, potent humoral immune responses were observed in an early clinical trial with repeated administration of T cells expressing a first‐generation CAR based on a murine scFv directed against carbonic anhydrase IX (CAIX) [78]. In this trial, the humoral responses were anti‐idiotypic in nature and neutralized the function of the CAIX‐specific CAR T cells. Notably, the occurrence of anti‐idiotypic antibodies in the blood coincided with the inability to detect circulating CAR T cells by anti‐idiotype antibody‐based flow cytometric analysis, whereas qPCR analysis of CAR DNA still showed their presence. In another study, repeated infusion of CAR T cells electroporated with mRNA coding for a CAR based on a murine scFv against human mesothelin resulted in anaphylaxis, likely caused by IgE antibodies directed against the CAR [87].

Given the lack of perfectly reliable models for the prediction of immunogenicity, it is generally desired to design therapeutic proteins that are as close to human germline as possible. For example, a chimeric antibody, in which the entire variable domains are of nonhuman origin, is considered more likely to be immunogenic than a humanized antibody which only comprises nonhuman CDRs and possibly some nonhuman framework positions. However, caution is needed, because the word 'humanized' is somewhat misleading, since it implies that the resulting monoclonal antibodies (mAbs) are human and therefore not distinguishable from an endogenous human protein. This is clearly not the case, given the nonhuman origin of their CDR loops. In addition, usually some framework mutations are needed to maintain binding and/or stability of the resulting humanized mAbs [88, 89].

**Fig. 5:**
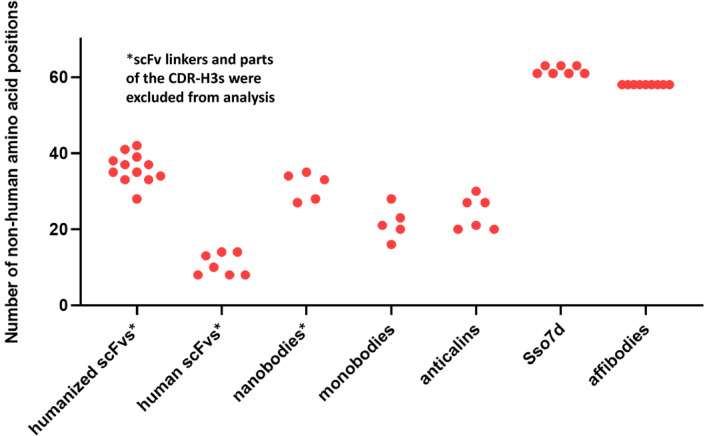
Analysis of the number of nonhuman amino acid positions in human and humanized scFvs as compared to engineered binding scaffolds based on human or nonhuman protein domains. To determine the deviation from human germline sequences for humanized and human scFvs, the V_H_ and V_L_ sequences of humanized and human blockbuster antibodies were obtained from the DrugBank (https://www.drugbank.ca/) and subsequently aligned to human antibody germline genes using the DomainGapAlign tool from IMGT (www.imgt.org). An alignment is exemplarily shown for trastuzumab in Fig. S1. Since the D germline segments could not be unambiguously assigned to the CDR‐H3 sequences, parts of the CDR‐H3s were omitted from analysis. Therefore, the actual number of nonhuman amino acid positions will be slightly higher for human and humanized scFvs, as well as for nanobodies, which were also aligned using the DomainGapAlign tool. To determine the number of nonhuman amino acid positions in engineered binding scaffolds, monobodies and anticalins were aligned against the original human proteins (FN3 domain and lipocalins, respectively), whereas Sso7d and affibodies were assumed to be 100% nonhuman. Since the charge‐reduced version of Sso7d (rcSso7d) is shortened by 2 amino acids, the amount of nonhuman positions is either 61 (rcSso7d‐based binders) or 63 (Sso7d‐based mutants). All names and sequences of analyzed proteins are depicted in Table S1.

Although the number of nonhuman amino acid positions is clearly not the only determinant of immunogenicity, we aimed at roughly comparing this deviation from human sequences in different antigen recognition domains. Therefore, we determined the number of amino acid positions differing from human germline‐encoded genes for the following antigen‐binding formats (Fig. 5): (i) scFvs derived from 'humanized' blockbuster antibodies (being defined here by worldwide sales of at least US$ 1 billion in 2016 [90]); (ii) scFvs derived from 'human' blockbuster antibodies; (iii) nanobodies based on camelid V_HH_ domains; (iv) monobodies based on the human FN3 domain; (v) anticalins based on human lipocalins; (vi) binders based on Sso7d or rcSso7d derived from *S. solfataricus*; and (vii) affibodies based on the Z‐domain of protein A from *S. aureus*. As expected, 'human' scFvs are closest to human germline sequences (Fig. 5). More surprisingly, engineered monobodies and anticalins tend to contain fewer nonhuman amino acid positions than humanized scFvs (Fig. 5). It should be noted that for the humanized and human scFvs (as well as for nanobodies) these numbers are probably underestimating the deviations from germline, due to four factors: First, we used the DomainGapAlign tool from IMGT (www.imgt.org), which was also recommended for alignment to human antibody germline sequences by the WHO INN Expert Group in April 2015 [88]. However, due to the short lengths of the D segments and additional junctional diversity and/or somatic hypermutation, it was not possible to assign those D segments to the CDR‐H3s. As a consequence, parts of the usually considerably mutated CDR‐H3 sequences were excluded from the analysis (Fig. S1), thereby reducing the number of nonhuman amino acid positions. Second, it is known that each individual only carries a fraction of the known germline antibody gene segments (e.g., approximately 50 out of 250 known V segments [91]). As a consequence, aligning an antibody against the full set of germline gene segments in the IMGT database will yield a closest match, which might not be present in a given patient. In other words, the closest V segment in that patient might differ even more from the given antibody sequence, which would further increase the number of nonhuman positions in that scFv. Third, it also needs to be taken into consideration that for proteins based on human germline genes (such as antibodies or monobodies), scattered mutations will result in novel nonhuman epitopes, even if these stretches only contain a certain percentage of mutated residues. As an example, a 12‐amino acid peptide containing six mutated residues does not look like a human peptide any more. Thus, while the entire peptide will be seen as 'nonhuman', the alignment only defines six mutated amino acid positions, thus again underestimating the extent of nonhuman sequence stretches. Fourth, the linkers between V_H_ and V_L_ in scFvs were also excluded from the analysis. Thus, the data for humanized and human scFvs and for nanobodies in Fig. 5 should be interpreted as conservative estimates.

Again, we would like to emphasize that the number of nonhuman amino acid positions in a protein is not expected to perfectly correlate with its immunogenic potential. Nevertheless, it can be expected that there is some trend. This was also the underlying assumption in the therapeutic antibody field, which moved from fully murine hybridoma antibodies to chimeric mAbs and finally to humanized and 'fully human' mAbs. Most importantly, the data in Fig. 5 clearly demonstrate that judging the risk of immunogenicity of a therapeutic protein solely based on its human vs. nonhuman origin would be an oversimplification and in many cases incorrect. For example, nanobodies derived from camelids tend to contain a comparable or even slightly lower number of nonhuman amino acid positions than humanized scFvs, which can be explained by the fact that—despite having more nonhuman framework positions—they only contain three CDR loops and are only approximately half the size of an scFv. Even binding scaffolds based on nonhuman proteins such as affibodies or Sso7d‐based binders only show slightly elevated levels of such nonhuman positions compared with humanized scFvs. Taking into consideration the expected underestimation of the deviation of scFvs from germline (as discussed above), the difference between humanized scFvs and engineered nonhuman binding scaffolds will be even smaller.

Finally, it should also be noted that in addition to foreign sequences within protein domains also the junctions between different CAR domains unavoidably create non‐native peptide stretches that may induce an immune response as has been observed, for example, with the oncogenic fusion protein Bcr‐Abl [92, 93].

## Natural receptors or ligands as alternatives to scFvs on CARs

Besides engineered binding scaffolds and scFvs, natural ligands and receptors can also be used as alternative binding moieties in CARs. They harbor several advantages compared with engineered protein scaffolds: (i) They naturally occur in the human body, thereby minimizing the risk of potential immunogenicity; (ii) many of them can bind multiple targets, which broadens the range of applications and prevents escape due to antigen downregulation; and (iii) some of their ligands are upregulated upon stress conditions and can thereby often be found in the tumor microenvironment.

### Natural killer group 2 member D‐CARs

One example for such a natural receptor is natural killer group 2 member D (NKG2D), a type II transmembrane protein present on various immune cells, such as natural killer (NK) cells, CD8^+^ T cells, and γδ T cells [94]. NKG2D binds to eight known ligands (MICA, MICB, and ULBP1 to ULBP6) which are present on a wide range of tumors of various categories, including carcinomas, sarcomas, leukemias, lymphomas, and multiple myelomas [95]. Notably, NKG2D ligands are not only expressed by tumor tissue, but can also be found on immunosuppressive cells such as regulatory T cells (Tregs) and myeloid‐derived suppressor cells (MDSCs) [96]. Given these expression patterns of NKG2D ligands, the extracellular domain of NKG2D is a promising candidate for an antigen recognition domain on a CAR. First reports of an NKG2D‐based CAR demonstrated efficacy in murine models and *in vitro* activity against human tumor cell lines [97, 98]. Lehner and colleagues developed an NKG2D‐CAR with a reengineered extracellular domain by fusing the C terminus of NKG2D in a type I membrane protein orientation to generate a second‐generation CAR with integrated CD28 costimulation [99]. Subsequently, NKG2D was fused to CAR backbones with 4‐1BB costimulation and—for expression of the CAR in NK cells—also to DAP12 [100, 101]. Meanwhile, NKG2D CAR T cells and also NKG2D CAR NK cells have already been tested in several clinical phase I studies [101, 102, 103]. Overall, the administration of NKG2D‐CAR cells in absence of lymphodepleting conditioning was well‐tolerated without significant side effects. Notably, despite limited persistence, promising clinical responses were observed in patients with AML and colorectal cancer [101, 103].

### Lymphocyte function‐associated antigen 1‐CARs

Another antigen that is commonly overexpressed by tumors is intercellular adhesion molecule 1 (ICAM1). It can be found on carcinomas but is also expressed at low level on endothelial and immune cells [104]. In previous studies, Park and colleagues have affinity‐matured the natural ligand, lymphocyte function‐associated antigen 1 (LFA‐1) by directed evolution to achieve several variants covering a huge affinity range (*K*_D_ 1 nm–1 mm) [105]. When these LFA‐1 variants with different affinities to ICAM1 were incorporated into a CAR comprising two costimulatory domains (CD28 and 4‐1BB), the authors found that both antigen density and affinity to ICAM1 were directly proportional to CAR T cell activity. Interestingly, CAR T cells incorporating high‐affinity binders not only killed tumor cells, but also induced systemic toxicity in a mouse model. In contrast, lower‐affinity CARs in the micromolar range could delay tumor growth without any observed toxicity. This study elegantly shows that natural ligands or receptors can even be affinity‐tuned by introducing a limited number of mutations (in this case only 1–3 mutations, depending on the LFA‐1 variant). The availability of recognition domains in a broad affinity range may be a critical advantage, allowing the investigator to choose the optimal affinity to yield highly efficacious CAR T cells, while only causing limited systemic toxicity.

### Interleukin 13 (IL‐13) mutein‐CARs

An attractive strategy to treat glioblastoma involves CAR T cells recognizing the IL‐13‐receptor α 2 (IL‐13Rα2), which is commonly overexpressed in brain tumors. In these CARs, antigen recognition is mediated by mutated versions of the antigen's natural ligand, that is, IL‐13 muteins. A recent case study reported a complete clinical response lasting for more than 7 months in a glioblastoma patient treated with repeated infusions of IL‐13 mutein CAR T cells [106]. Together, IL‐13 muteins represent a further example of natural ligands, which have been engineered through introduction of a limited number of mutations to achieve a therapeutically optimal affinity.

## Conclusion

Due to the availability of monoclonal antibodies against virtually any antigen, scFvs represent a convenient option for the design of CARs. However, many scFvs have been shown to trigger tonic CAR signaling and T cell exhaustion [16, 18, 20], presumably caused by scFv clustering. As extensively discussed in this review, there is no biochemical or functional requirement for the use of scFvs, as demonstrated by multiple studies using engineered binding scaffolds or natural ligands or receptors for the construction of CARs. Therefore, we anticipate that these alternative antigen recognition domains will be increasingly used in the CAR field, hopefully overcoming some of the current challenges in CAR T cell therapy.

## Conflicts of interest

C.U.Z., B.S., M.L., and M.W.T. have filed patent applications related to the technologies described in this manuscript. The other authors declare no conflict of interest.

## Author contributions

JMT and STR performed sequencing alignments. CUZ and MWT analyzed the sequencing alignments to yield mutation counts. CUZ, BS, ML, and MWT wrote the manuscript. All authors read and edited the manuscript.

## Supporting information


**Fig. S1.** Representative V_H_ and V_L_ alignments. V_H_ and V_L_ sequences of human and humanized antibodies were obtained from the DrugBank (https://www.drugbank.ca/) and aligned against human antibody germline sequences using the DomainGapAlign tool from IMGT (www.imgt.org).
**Table S1.** Amino acid sequences of V_H_ and V_L_ domains derived from human and humanized antibodies and engineered binding scaffolds used for the analysis of the number of non‐human amino acid positions in Figure 5.
