# Supplementary material for: Driving CARs with alternative navigation tools – the potential of engineered binding scaffolds
Source: FEBS J. 2020 Aug 31;288(7):2103–18. doi: 10.1111/febs.15523 (PMC8048499; doi:10.1111/febs.15523)
Supplement: Supplementary file 1 — Fig. S1. Representative VH and VL alignments. VH and VL sequences of human and humanized antibodies were obtained from the DrugBank (https://www.drugbank.ca/) and aligned against human antibody germline sequences using the DomainGapAlign tool from IMGT (www.imgt.org). Table S1. Amino acid sequences of VH and VL domains derived from human and humanized antibodies and engineered binding scaffolds used for the analysis of the number of non‐human amino acid positions in Figure 5. [file FEBS-288-2103-s001.zip › febs15523-sup-0001-Supinfo.pdf]

FEBS PRESS
science publishing by scientists

WILEY

The FEBS Journal

www.febsjournal.org

Driving CARs with alternative navigation tools – the potential of engineered binding scaffolds

Charlotte U. Zajc, Benjamin Salzer, Joseph M. Taft, Sai T. Reddy, Manfred Lehner and Michael W. Traxlmayr

DOI: 10.1111/febs.15523

Supplemental Figure 1

Alignment of the V_L domain of trastuzumab (Herceptin)

```
            FR1-IMGT              CDR1-IMGT     FR2-IMGT        CDR2-IMGT                 FR3-IMGT                  CDR3-IMGT    FR4-IMGT
             (1-26)               (27-38)       (39-55)         (56-65)                   (66-104)                  (105-117)    (118-128)
         A               B          BC          C         C'       C'C"         C"        D            E          F          FG           G
      (1-15)          (16-26)    (27-38)     (39-46)   (47-55)   (56-65)     (66-74)   (75-84)      (85-96)    (97-104)   (105-117)    (118-128)
 --------------->  ------------>             -------->  -------->            -------->  ---------->  ----------->  -------->            ------------>
 1         10   15 16     23 26 27         38 3941   46 47      55 56       65 66      74 75      84 85  89    96 97   104 105 11112 117 118       128
 |........|....|  |......|..|  |..........|  |.|....|  |.......|  |........|  |.......|  |.........|  |...|......|  |......|  |.....||....|  |.........|

Herceptin_VL    DIQMTQSPSSLSASV GDRVTITCRAS QDV......NTA VAWYQQKP GKAPKLLIY SA.......S FLYSGVP.S RFSGSR..SG TDFTLTISSLQP EDFATYYC QQHYT....TPPT FGQGTKVEIK.

IGKV1-39*01     DIQMTQSPSSLSASV GDRVTITCRAS QSI......SSY LNWYQQKP GKAPKLLIY AA.......S SLQSGVP.S RFSGSG..SG TDFTLTISSLQP EDFATYYC QQSYSTP
Homo sapiens                                DV       NTA VA                  S          F Y          R                                   H T

                                                                                                                                     WT FGQGTKVEIK    IGKJ1*01
                                                                                                                                     P                Homo sapiens
```

Alignment of the V_H domain of trastuzumab (Herceptin)

```
            FR1-IMGT              CDR1-IMGT     FR2-IMGT        CDR2-IMGT                 FR3-IMGT                  CDR3-IMGT    FR4-IMGT
             (1-26)               (27-38)       (39-55)         (56-65)                   (66-104)                  (105-117)    (118-128)
         A               B          BC          C         C'       C'C"         C"        D            E          F          FG           G
      (1-15)          (16-26)    (27-38)     (39-46)   (47-55)   (56-65)     (66-74)   (75-84)      (85-96)    (97-104)   (105-117)    (118-128)
 --------------->  ------------>             -------->  -------->            -------->  ---------->  ----------->  -------->            ------------>
 1         10   15 16     23 26 27         38 3941   46 47      55 56       65 66      74 75      84 85  89    96 97   104 105 11112 117 118       128
 |........|....|  |......|..|  |..........|  |.|....|  |.......|  |........|  |.......|  |.........|  |...|......|  |......|  |.....||....|  |.........|

Herceptin_VH    EVQLVESGG.GLVQP GGSLRLSCAAS GFNI....KDTY IHWVRQAP GKGLEWVAR IYPT..NGYT RYADSVK.G RFTISADTSK NTAYLQMNSLRA EDTAVYYC SRWGGDGFYAMDY WGQGTLVTVSS

IGHV3-66*01     EVQLVESGG.GLVQP GGSLRLSCAAS GFTV....SSNY MSWVRQAP GKGLEWVSV IYSG...GST YYADSVK.G RFTISRDNSK NTLYLQMNSLRA EDTAVYYC AR
Homo sapiens                                NI      KDT IH            AR     PT    Y R                A T      A                   S

                                                                                                                                YFDY WGQGTLVTVSS    IGHJ4*01
                                                                                                                                AM                  Homo sapiens
```

Humanized antibodies

References: https://www.drugbank.ca/

Supplemental Table 1

Name	Trade name	Class	Target
**Trastuzumab**	Herceptin	IgG1	Her2

V_H sequence

EVQLVESGGGLVQPGGSLRLSCAASGFNIKDTYIHWVRQAPGKGLEWVARIYPTNGYTRYADSVKGRFTISADTSKNTAYLQMNSLRAEDTAVYYCSRWGGDGFYAMDYWGQGTLVTVSS

V_L sequence

DIQMTQSPSSLSASVGDRVTITCRASQDVNTAVAWYQQKPGKAPKLLIYSASFLYSGVPSRFSGSRSGTDFTLTISSLQPEDFATYYCQQHYTTPPTFGQGTKVEIK

Name	Trade name	Class	Target
**Pertuzumab**	Perjeta	IgG1	Her2

V_H sequence

EVQLVESGGGLVQPGGSLRLSCAASGFTFTDYTMDWVRQAPGKGLEWVADVNPNSGGSIYNQRFKGRFTLSVDRSKNTLYLQMNSLRAEDTAVYYCARNLGPSFYFDYWGQGTLVTVSS

V_L sequence

DIQMTQSPSSLSASVGDRVTITCKASQDVSIGVAWYQQKPGKAPKLLIYSASYRYTGVPSRFSGSGSGTDFTLTISSLQPEDFATYYCQQYYIYPYTFGQGTKVEIK

Name	Trade name	Class	Target
**Pembrolizumab**	Keytruda	IgG4	PD-1

V_H sequence

QVQLVQSGVEVKKPGASVKVSCKASGYTFTNYYMYWVRQAPGQGLEWMGGINPSNGGTNFNEKFKNRVTLTTDSSTTTAYMELKSLQFDDTAVYYCARRDYRFDMGFDYWGQGTTVTVSS

V_L sequence

EIVLTQSPATLSLSPGERATLSCRASKGVSTSGYSYLHWYQQKPGQAPRLLIYLASYLESGVPARFSGSGSGTDFTLTISSLEPEDFAVYYCQHSRDLPLTFGGGTKVEIK

Name	Trade name	Class	Target
**Bevacizumab**	Avastin	IgG1	VEGF

V_H sequence

EVQLVESGGGLVQPGGSLRLSCAASGYTFTNYGMNWVRQAPGKGLEWVGWINTYTGEPTYAADFKRRFTFSLDTSKSTAYLQMNSLRAEDTAVYYCAKYPHYYGSSHWYFDVWGQGTLVTVSS

V_L sequence

DIQMTQSPSSLSASVGDRVTITCSASQDISNYLNWYQQKPGKAPKVLIYFTSSLHSGVPSRFSGSGSGTDFTLTISSLQPEDFATYYCQQYSTVPWTFGQGTKVEIK

Name	Trade name	Class	Target
**Natalizumab**	Tysabri	IgG4	a4b1 and a4b7 integrins

V_H sequence

QVQLVQSGAEVKKPGASVKVSCKASGFNIKDTYIHWVRQAPGQRLEWMGRIDPANGYTKYDPKFQGRVTITADTSASTAYMELSSLRSEDTAVYYCAREGYYGNYGVYAMDYWGQGTLVTVSS

V_L sequence

DIQMTQSPSSLSASVGDRVTITCKTSQDINKYMAWYQQTPGKAPRLLIHYTSALQPGIPSRFSGSGSGRDYTFTISSLQPEDIATYYCLQYDNLWTFGQGTKVEIK

Name	Trade name	Class	Target
**Vedolizumab**	Entyvio	IgG1	a4b7 integrin

V_H sequence

QVQLVQSGAEVKKPGASVKVSCKGSGYTFTSYWMHWVRQAPGQRLEWIGEIDPSESNTNYNQKFKGRVTLTVDISASTAYMELSSLRSEDTAVYYCARGGYDGWDYAIDYWGQGTLVTVSS

V_L sequence

DVVMTQSPLSLPVTPGEPASISCRSSQSLAKSYGNTYLSWYLQKPGQSPQLLIYGISNRFSGVPDRFSGSGSGTDFTLKISRVEAEDVGVYYCLQGTHQPYTFGQGTKVEIK

Name	Trade name	Class	Target
**Omalizumab**	Xolair	IgG1	IgE

V_H sequence

EVQLVESGGGLVQPGGSLRLSCAVSGYSITSGYSWNWIRQAPGKGLEWVASITYDGSTNYADSVKGRFTISRDDSKNTFYLQMNSLRAEDTAVYYCARGSHYFGHWHFAVWGQGTLVTVSS

V_L sequence

DIQLTQSPSSLSASVGDRVTITCRASQSVDYDGDSYMNWYQQKPGKAPKLLIYAASYLESGVPSRFSGSGSGTDFTLTISSLQPEDFATYYCQQSHEDPYTFGQGTKVEIK

Name	Trade name	Class	Target
**Tocilizumab**	Actemra	IgG1	IL-6 R

V_H sequence

QVQLQESGPGLVRPSQTLSLTCTVSGYSITSDHAWSWVRQPPGRGLEWIGYISYSGITTYNPSLKSRVTMLRDTSKNQFSLRLSSVTAADTAVYYCARSLARTTAMDYWGQGSLVTVSS

V_L sequence

DIQMTQSPSSLSASVGDRVTITCRASQDISSYLNWYQQKPGKAPKLLIYYTSRLHSGVPSRFSGSGSGTDFTFTISSLQPEDIATYYCQQGNTLPYTFGQGTKVEIK

Name	Trade name	Class	Target
**Certolizumab**	Cimzia	IgG1	TNF

V_H sequence

EVQLVESGGGLVQPGGSLRLSCAASGYVFTDYGMNWVRQAPGKGLEWMGWINTYIGEPIYADSVKGRFTFSLDTSKSTAYLQMNSLRAEDTAVYYCARGYRSYAMDYWGQGTLVTVSS

V_L sequence

DIQMTQSPSSLSASVGDRVTITCKASQNVGTNVAWYQQKPGKAPKALIYSASFLYSGVPYRFSGSGSGTDFTLTISSLQPEDFATYYCQQYNIYPLTFGQGTKVEIK

Name	Trade name	Class	Target
**Eculizumab**	Soliris	IgG2/4	C5

V_H sequence

QVQLVQSGAEVKKPGASVKVSCKASGYIFSNYWIQWVRQAPGQGLEWMGEILPGSGSTEYTENFKDRVTMTRDTSTSTVYMELSSLRSEDTAVYYCARYFFGSSPNWYFDVWGQGTLVTVSS

V_L sequence

DIQMTQSPSSLSASVGDRVTITCGASENIYGALNWYQQKPGKAPKLLIYGATNLADGVPSRFSGSGSGTDFTLTISSLQPEDFATYYCQNVLNTPLTFGQGTKVEIK

Name	Trade name	Class	Target
**Palivizumab**	Synagis	IgG1	RSV gpF

V_H sequence

QVTLRESGPALVKPTQTLTLTCTFSGFSLSTSGMSVGWIRQPPGKALEWLADIWWDDKKDYNPSLKSRLTISKDTSKNQVVLKVTNMDPADTATYYCARSMITNWYFDVWGAGTTVTVSS

V_L sequence

DIQMTQSPSTLSASVGDRVTITCKCQLSVGYMHWYQQKPGKAPKLLIYDTSKLASGVPSRFSGSGSGTAFTLTISSLQPDDFATYYCFQGSGYPFTFGGGTKLEIK

Name	Trade name	Class	Target
**Ranibizumab**	Lucentis	IgG1	VEGF

V_H sequence

EVQLVESGGGLVQPGGSLRLSCAASGYDFTHYGMNWVRQAPGKGLEWVGWINTYTGEPTYAADFKRRFTFSLDTSKSTAYLQMNSLRAEDTAVYYCAKYPYYYGTSHWYFDVWGQGTLVTVSS

V_L sequence

DIQLTQSPSSLSASVGDRVTITCSASQDISNYLNWYQQKPGKAPKVLIYFTSSLHSGVPSRFSGSGSGTDFTLTISSLQPEDFATYYCQQYSTVPWTFGQGTKVEIK

Human antibodies

References: https://www.drugbank.ca/

Name	Trade name	Class	Target
**Ipilimumab**	Yervoy	IgG1	CTLA4

V_H sequence

QVQLVESGGGVVQPGRSLRLSCAASGFTFSSYTMHWVRQAPGKGLEWVTFISYDGNNKYYADSVKGRFTISRDNSKNTLYLQMNSLRAEDTAIYYCARTGWLGPFDYWGQGTLVTVSS

V_L sequence

EIVLTQSPGTLSLSPGERATLSCRASQSVGSSYLAWYQQKPGQAPRLLIYGAFSRATGIPDRFSGSGSGTDFTLTISRLEPEDFAVYYCQQYGSSPWTFGQGTKVEIK

Name	Trade name	Class	Target
**Nivolumab**	Opdivo	IgG4	PD1

V_H sequence

QVQLVESGGGVVQPGRSLRLDCKASGITFSNSGMHWVRQAPGKGLEWVAVIWYDGSKRYYADSVKGRFTISRDNSKNTLFLQMNSLRAEDTAVYYCATNDDYWGQGTLVTVSS

V_L sequence

EIVLTQSPATLSLSPGERATLSCRASQSVSSYLAWYQQKPGQAPRLLIYDASNRATGIPARFSGSGSGTDFTLTISSLEPEDFAVYYCQQSSNWPRTFGQGTKVEIK

Name	Trade name	Class	Target
**Denosumab**	Prolia/Xgeva	IgG2	RANKL

V_H sequence

EVQLLESGGGLVQPGGSLRLSCAASGFTFSSYAMSWVRQAPGKGLEWVSGITGSGGSTYYADSVKGRFTISRDNSKNTLYLQMNSLRAEDTAVYYCAKDPGTTVIMSWFDPWGQGTLVTVSS

V_L sequence

EIVLTQSPGTLSLSPGERATLSCRASQSVRGRYLAWYQQKPGQAPRLLIYGASSRATGIPDRFSGSGSGTDFTLTISRLEPEDFAVFYCQQYGSSPRTFGQGTKVEIK

Name	Trade name	Class	Target
**Ustekinumab**	Stelara	IgG1	IL-2 and IL-23

V_H sequence

EVQLVQSGAEVKKPGESLKISCKGSGYSFTTYWLGWVRQMPGKGLDWIGIMSPVDSDIRYSPSFQGQVTMSVDKSITTAYLQWNSLKASDTAMYYCARRRPGQGYFDFWGQGTLVTVSS

V_L sequence

DIQMTQSPSSLSASVGDRVTITCRASQGISSWLAWYQQKPEKAPKSLIYAASSLQSGVPSRFSGSGSGTDFTLTISSLQPEDFATYYCQQYNIYPYTFGQGTKLEIK

Name	Trade name	Class	Target
**Secukinumab**	Cosentyx	IgG1	IL-17A

V_H sequence

EVQLVESGGGLVQPGGSLRLSCAASGFTFSNYWMNWVRQAPGKGLEWVAAINQDGSEKYYVGSVKGRFTISRDNAKNSLYLQMNSLRVEDTAVYYCVRDYYDILTDYYIHYWYFDLWGRGTLVTVSS

V_L sequence

EIVLTQSPGTLSLSPGERATLSCRASQSVSSSYLAWYQQKPGQAPRLLIYGASSRATGIPDRFSGSGSGTDFTLTISRLEPEDFAVYYCQQYGSSPCTFGQGTRLEIK

Name	Trade name	Class	Target
**Adalimumab**	Humira	IgG1	TNF

V_H sequence

EVQLVESGGGLVQPGRSLRLSCAASGFTFDDYAMHWVRQAPGKGLEWVSAITWNSGHIDYADSVEGRFTISRDNAKNSLYLQMNSLRAEDTAVYYCAKVSYLSTASSLDYWGQGTLVTVSS

V_L sequence

DIQMTQSPSSLSASVGDRVTITCRASQGIRNYLAWYQQKPGKAPKLLIYAASTLQSGVPSRFSGSGSGTDFTLTISSLQPEDVATYYCQRYNRAPYTFGQGTKVEIK

Name	Trade name	Class	Target
**Golimumab**	Simponi	IgG1	TNF

V_H sequence

QVQLVESGGGVVQPGRSLRLSCAASGFIFSSYAMHWVRQAPGNGLEWVAFMSYDGSNKKYADSVKGRFTISRDNSKNTLYLQMNSLRAEDTAVYYCARDRGIAAGGNYYYYGMDVWGQGTTVTVSS

V_L sequence

EIVLTQSPATLSLSPGERATLSCRASQSVYSYLAWYQQKPGQAPRLLIYDASNRATGIPARFSGSGSGTDFTLTISSLEPEDFAVYYCQQRSNWPPFTFGPGTKVDIK

Nanobodies

Scaffold	Variant name	Target	Reference
VHH	VHH-D4	fluorescin	https://doi.org/10.3390/ijms19113444

Sequence

QVQLVESGGALVQPGGSLRLSCAASGFTFGSFSMSWYRQATGKEREWVAGLSAGSSSTYYADSVKGRFTISRDNARNTVYLQMNSLKPEDTAVYYCARRTNTSSGSAGSFSTCIDDWGQGTQVTVSS

Scaffold	Variant name	Target	Reference
VHH	Nb5	Gβγ dimer	https://doi.org/10.1038/s41467-018-04432-0

Sequence

QVQLVESGGGLVQAGGSLRLSCAASGSIFSINAMGWYRQAPGKQRELVAAITRGGRTNYADSVKGRFTLSRDNAKNTVYLQMNSLKPEDTAVYYCNVGRSRGYWGQGTQVTVSS

Scaffold	Variant name	Target	Reference
VHH	VHH-28	gp140 trimer	https://doi.org/10.1038/s41598-017-08273-7

Sequence

DVQLQESGGGSVQAGGSLRLSCVASGVTSTRPCIGWFRQAPGKEREGVAVVNFRGDSTYITDSVKGRFTISRDEDSDTVYLQMNSLKPEDTATYYCAADVNRGGFCYIEDWYFSYWGQGTQVTVSS

Scaffold	Variant name	Target	Reference
VHH	aCaffVHH	Caffeine/aCaffVHH	https://doi.org/10.1038/s41467-018-04744-1

Sequence

QVQLVESGGGLVQAGGSLRLSCTASGRTGTIYSMAWFRQAPGKEREFLATVGWSSGITYYMDSVKGRFTISRDKGKNTVYLQMDSLKPEDTAVYYCTATRAYSVGYDYWGQGTQVTVSS

Scaffold	Variant name	Target	Reference
VHH	1053	CD38	doi: 10.1038/srep27055

Sequence

DVQLQESGGGLVQAGGSLRLSCTGSGRTFRNYPMAWFRQAPGKEREFVAGITWVGASTLYADFAKGRFTISRDNAKNTVYLQMNSLKPEDTAVYSCAAGRGIVAGRIPAEYADWGQGTQVTVSS

Anticalins

Scaffold	Variant name	Target	Reference
**Lcn2**	WT		

Sequence

QDSTSDLIPAPPLSKVPLQQNFQDNQFQGKWYVVGLAGNAILREDKDPQKMYATIYELKEDKSYNVTSVLFRKKKCDYWIRTFVPGCQPGEFTLGNIKSYPGLTSYLVRVVSTNYNQHAMVFFKKVSQN

Sequence (continued)

REYFKITLYGRTKELTSELKENFIRFSKSLGLPENHIVFPVPIDQCIDG

Scaffold	Variant name	Target	Reference
**Lcn2**	U3D7	VEGFR-3	https://doi.org/10.1515/hsz-2016-0195

Sequence

QDSTSDLIPAPPLSKVPLQQNFQDNQFHGKWYVVGTAGNRRLREDKDPPKMSATIYELKEDKSYNVTVVWFWKKKCEYNIDTFVPGSQPGEFTLGSIKSEPGQTSILVRVVSTNYNQHAMVFFKTVDQ

Sequence (continued)

NREAFAITLYGRTKELTSELKENFIRFSKSLGLPENHIVFPVPIDQCIDG

Scaffold	Variant name	Target	Reference
**Lcn2**	A3A5	PSMA	https://doi.org/10.1093/protein/gzv065

Sequence

QDSTSDLIPAPPLSKVPLQQNFQDNQFHGKWYVVGLAGNVILREDKDPYKMSATIYELKEDKSYNVTNVRFYLKKCYYTIATFVPGSQPGEFTLGTIKSGPGKTSGLVRVVSTNYNQHAMVFFKEVQQN

Sequence (continued)

REWFIITLYGRTKELTSELKENFIRFSKSLGLPENHIVFPVPIDQCIDG

Scaffold	Variant name	Target	Reference
**Lcn2**	#003	CTLA-4	doi: 10.1073/pnas.0813399106

Sequence

QDSTSDLIPAPPLSKVPLQQNFQDNQFHGKWYVVGLAGNRILRDDQHPMNMYATIYELKEDKSYNVTSVISSHKKCEYTIATFVPGSQPGEFTLGNIKSYGDKTSYLVRVVSTDYNQYAVVFFKLAEDNA

Sequence (continued)

EFFAITIYGRTKELASELKENFIRFSKSLGLPENHIVFPVPIDQCIDG

**Scaffold**	**Variant name**	**Target**	**Reference**
**Lcn2**	N7A	FN3	https://doi.org/10.1016/j.jmb.2012.12.004

Sequence

QDSTSDLIPAPPLSKVPLQQNFQDNQFHGKWYVVGKAGNHDLREDKDPRKMQATIYELKEDKSYNVTNVRFVHKKCNYRIWTFVPGSQPGEFTLGNIKSWPGLTSWLVRVVSTNYNQHAMVFFKRV

Sequence (continued)

YQNRELFEITLYGRTKELTNELKENFIRFSKSLGLPENHIVFPVPIDQCIDG

**Scaffold**	**Variant name**	**Target**	**Reference**
**Lcn1/Tlc**	WT		

Sequence

ASDEEIQDVSGTWYLKAMTVDREFPEMNLESVTPMTLTTLEGGNLEAKVTMLISGRCQEVKAVLEKTDEPGKYTADGGKHVAYIIRSHVKDHYIFYCEGELHGKPVRGVKLVGRDPKNNLEALEDFEKAAG

Sequence (continued)

ARGLSTESILIPRQSETCSPG

**Scaffold**	**Variant name**	**Target**	**Reference**
**Lcn1/Tlc**	PRS-050	VEGF-A	https://doi.org/10.1371/journal.pone.0083232

Sequence

ASDEEIQDVSGTWYLKAMTVDVGALRCLAGSVIPTTLTTLEGGNLEAKVTMHIKGRSQEVKAVLSKTDEPGIYTAIGGIHVAKIGRSHVKDHYIFYSEGCLSGVPVPGVWLVGRDPKNNLEALEDFEKAAGA

Sequence (continued)

RGLSTESILIPRQSETSSPG

**Scaffold**	**Variant name**	**Target**	**Reference**
**Lcn1/Tlc**	S191.4-B24	IL4-Ralpha	WO2011/154420

Sequence

ASDEEIQDVSGTWYLKAMTVDSRCPRAYYSSVTPMTLTLLEGGNLEAKFTAQRSGRWQEYKLVLEKTDEPGKYTASGGRHVAYIIRSHVKDHYIFHSEGLCPGQPVPGVWLVGRDPKNNLEALEDFEKAA

Sequence (continued)

GARGLSTESILIPRQSETSSPG

Monobodies

Scaffold	Variant name	Target	Reference
**FN3**	WT		

Sequence

VSDVPRDLEVVAATPTSLLISWDAPAVTVRYYRITYGETGGNSPVQEFTVPGSKSTATISGLKPGVDYTITVYAVTGRGDSPASSKPISINYRT

Scaffold	Variant name	Target	Reference
**FN3**	RF2	hRBP4 + A1120	https://doi.org/10.1073/pnas.1911154117

Sequence

VSDVPRDLEVVAATPTSLLISWYYPNASHAGYYRITYGETGGNSPVQEFTVPFSIRYTIATISGLKPGVDYTITVYAVTDYAYYYRLSEPISINYRT

Scaffold	Variant name	Target	Reference
**FN3**	NSa1	SHP2	https://doi.org/10.1073/pnas.1303640110

Sequence

VSSVPTKLEVVAATPTSLLISWDAPAVTVDYYVITYGETGSGGYAWQEFEVPGSKSTATISGLKPGVDYTITVYAGYYGYPTYYSSPISINYRT

Scaffold	Variant name	Target	Reference
**FN3**	ySMB-9	SUMO1	doi:10.1016/j.jmb.2011.12.019.

Sequence

VSSVPTKLEVVAATPTSLLISWDAGYWFIDYYRITYGETGGNSPVQEFTVPGYSSTATISGLSPGVDYTITVYAYYDNYGWSPISINYRT

Scaffold	Variant name	Target	Reference
**FN3**	7c12	SH2	https://doi.org/10.1016/j.cell.2011.08.046

Sequence

VSSVPTKLEVVDATPTSLKISWDAYYSSWQNVKYYRITYGETGGDSPVQEFTVPGYYSTATISGLKPGVDYTITVYAYDTFFPGYEPNSPISINYRT

Scaffold	Variant name	Target	Reference
**FN3**	I-Lead	IFG-IR	doi: 10.4161/mabs.3.1.14168

Sequence

VSDVPRDLEVVAATPTSLLISWSARLKVARYYRITYGETGGNSPVQEFTVPKNVYTATISGLKPGVDYTITVYAVTRFRDYQYPISINYRT

Sso7d

Scaffold	Variant name	Target	Reference
**Sso7d**	WT		

Sequence

ATVKFTYQGEEKQVDISKIKWVIRWGQHIAFKYDEGGGAAGYGWVSEKDAPKELLQMLEKQKK

Scaffold	Variant name	Target	Reference
**Sso7d**	M11.2	MSA	doi: 10.1074/jbc.M116.741314

Sequence

ATVKFTYQGEEKQVDISKIKWVNRWGQRIYFKYDEGGGAAGYGWVSEKDAPKELLQMLEKQ

Scaffold	Variant name	Target	Reference
**Sso7d**	E18.1	EGFR	doi: 10.1074/jbc.M116.741314

Sequence

ATVKFTYQGEEKQVDISKIKWVIRLGRTIMFKYDEGGGANGYGKVSEKDAPKELLQMLEKQ

Scaffold	Variant name	Target	Reference
**Sso7d**	RS3	hRBP4 + A1120	https://doi.org/10.1073/pnas.1911154117

Sequence

ATVKLTYQGEEKQVDISKIKRVARYGQNIYFSYDEGGGAYDYGAVSEKDAPKELLQMLEKQ

Scaffold	Variant name	Target	Reference
**Sso7d**	N9	Notch1	doi: 10.1038/s41598-017-12246-1

Sequence

ATVKFYKGEEKQCDISAIIKVLRAGKNILFKYDLGGGKRGSGYVSEKDAPKELLQMLEKQKK

Scaffold	Variant name	Target	Reference
**Sso7d**	Sso7d-mIgG	mIgG	https://doi.org/10.1016/j.jmb.2011.04.020

Sequence

ATVKFKYKGEEKCVDISKIYLVLRLGKFIYFYYDLGGGKLGLGHVSEKDAPKELLQMLEKQKK

Scaffold	Variant name	Target	Reference
**Sso7d**	Sso7d-fluorescin	Sso7d-fluorescin	https://doi.org/10.1016/j.jmb.2011.04.020

Sequence

ATVKFKYKGEEKCVDISKIFRVIRSGKAIRFLYDLGGGKFGYGVVSEKDAPKELLQMLEKQKK

Scaffold	Variant name	Target	Reference
**Sso7d**	R11.1.1	K-Ras	https://doi.org/10.1038/s41598-017-05889-7

Sequence

ATVKFTYQGEEKQVDISKIKWVIRWGRYIWFKYDEDGGAKGWGYVSEKDAPKELLQMLGKQ

Affibodies

Scaffold	Variant name	Target	Reference
**Protein A**	WT		

Sequence

VDNKFNEQQNAFYEILHLPNLNEEQRNAFIQSLKDDPSQSANLLAEKKLNDAQAPK

Scaffold	Variant name	Target	Reference
**Protein A**	2B87	taq polymerase	DOI: 10.1016/j.jmb.2006.04.043

Sequence

VDNKFNKELGWATWEIFNLPNLNGVQVKAFIDSLRDDPSQSANLLAEAKKLNDAQAPK

Scaffold	Variant name	Target	Reference
**Protein A**	Ztaq:1154	taq polymerase	https://patents.google.com/patent/US9469670

Sequence

VDNKFNKEKGEAVVEIFRLPNLNGRQVKAFIASLYDDPSQSANLLAEAKKLNDAQAPK

Scaffold	Variant name	Target	Reference
**Protein A**	3MZW	HER2	DOI: 10.1073/pnas.1005025107

Sequence

VDNKFNKEMRNAYWEIALLPNLNNQQKRAFIRSLYDDPSQSANLLAEAKKLNDAQAPK

Scaffold	Variant name	Target	Reference
**Protein A**	ZHER2:342	HER2	https://patents.google.com/patent/US9469670

Sequence

VDNKFNKEMRNAYWEIALLPNLNNQQKRAFIRSLYDDPSQSANLLAEAKKLNDAQAPK

Scaffold	Variant name	Target	Reference
**Protein A**	ZEGFR:1907	EGFR	https://doi.org/10.1016/j.jmb.2007.12.060

Sequence

VDNKFNKEMWAAWEEIRNLPNLNGWQMTAFIASLVDDPSQSANLLAEAKKLNDAQAPK

Scaffold	Variant name	Target	Reference
**Protein A**	ZIGF1R:4551	IGF-1R	https://patents.google.com/patent/US8426557?oq=affibody%2binsulin

Sequence

VDNKFNKEGFYAALEILILPNLTQKQRGAFISSLSDDPSQSANLLAEAKKLNDAQAPK

Scaffold	Variant name	Target	Reference
**Protein A**	ZTNF:185	TNF	doi:10.1042/BA20090085

Sequence

VDNKFNKELGWAIGEIGTLPNLNHQQFRAFILSLWDDPSQSANLLAEAKKLNDAQAPK

Scaffold	Variant name	Target	Reference
**Protein A**	Z02465	PDGFRb	doi:10.1016/j.jmb.2011.01.033

Sequence

VDNKFNKELIEAAAEIDALPNLNRRQWNAFIKSLVDDPSQSANLLAEAKKLNDAQAPK

Scaffold	Variant name	Target	Reference
**Protein A**	Zinsulin:810	Insulin	https://patents.google.com/patent/US9469670

Sequence

VDNKFNKEKYMAYGEIRLLPNLNHQQVMAFIDSLVDDPSQSANLLAEAKKLNDAQAPK
